# Botany, in Its Relations with Medicine—No. 3

**Published:** 1877-12

**Authors:** W. T. Grant

**Affiliations:** Atlanta, Ga.


### BOTANY IN ITS RELATIONS WITH MEDICINE—No. 3.

By W. T. Grant, M.D., Atlanta, Ga.

It cannot be amiss to give a short explanation of the rule which obtains in reference to botanical nomenclature. In botany, as in other branches of science, the names are all double—the first being the *generic*, and the second the *specific* name. The generic name is, we may say, the *family* name, just as it is with the Smiths, the Joneses, etc., while the specific name is the personal or individual title, like the given name in a family, as John, William or Mary. We have, for instance, a family of Smiths consisting of John, Thomas, Joseph, and Mary Smith. All of them bear the common family name of Smith, as it is the family name. But each one has another and individual name, by which each is known and distinguished. So in botany, the generic name is the family name, common to several distinct species, each one of which has likewise its distinctive individual name, which is its *specific* title. Thus we have the genus solanum, and under it are the several species: solanum nigrum, s. tuberosum, s. carolinianum, etc. And, as in the family name among men there is a connection between the different individuals composing the family, so there is between the different species of a genus, but of course in a different way. It is blood-relationship in the family, and resemblance, more or less close, in the species.

As in society we find that communities are made up of several families, so in botany we find that several genera, more or less connected with each other by resemblances, are classed together, and the group thus formed, is designated a *natural order*. As we will find that the village of Richland is composed of the several families of Smiths, Joneses, Jacksons, Williams, etc., and as thus the individuals of these families may properly be called Richlanders, so with the different species forming the genera which together go to make up any natural order in botany. As, for instance, in the natural order *ranunculaceæ* we have the several genera, *aquilegia, ranunculus,* etc. Every species under these genera is called a ranunculad, or, as the common name is better known, is called a crowfoot.

The generic name in botany has no certain origin, but is mostly, I believe, given from men's names—generally he who gives an accurate description of a new genus has his name conferred upon it. The specific names are variously derived, but are most generally founded upon some characteristic of the plant, and most commonly upon the leaf.

It is a common opinion that because a plant is aromatic in odor, or pungent to the taste, it therefore possesses some valuable medical properties. My observation of plants leads me to the conclusion that the reverse is more apt to be true. These aromatic plants have their cells filled with aromatic oils, which are generally as innocent of any really valuable medical properties as the volatile or essential oils of the shops, which are only useful as a vehicle or disguise for other remedies. *Stomachics* they are magniloquently called, and it is a great pity that these grand names do not confer some virtue.

If it were true that plants possessed medical virtues *because* they are aromatic or pungent, then a large part of the plants belonging to *leguminosæ, labiatæ scrophulariaceæ,* and other allied natural orders, would be available. Besides these, the entire class of *umbelifers,* nearly every one of which is poisonous, would in that case furnish a valuable resource to the physician. But it is otherwise. And I do not now remember any really valuable plant which possesses aromatic properties.

The old and well-known *krameria lanceolata* (the rhatany of the shops) is a native of Georgia, but so far as I know, does not grow anywhere except on the left bank of the Little

Ocmulgee river, in Montgomery county. I need not speak of its properties. The restricted limits within which some plants grow, (as in this instance) is quite remarkable. Although I have botanized in nearly all parts of Georgia, I have never found the American cowslip, except in one locality—and then but a few individuals at that—in Crawford county. I know of others which have such limited range.

Another old acquaintance, the *chimaphila maculata*, grows quite abundantly in Clarke, Madison, Jackson, Hall, and other eastern counties, where it is popularly called wild arsenic. It is also found in Fulton county, where it passes under the name of wild ratsbane. This plant is blessed with several common names, as follows: spotted wintergreen, princes' pine, and pipsissewa.

I have had a limited experience with the chimaphila maculata in dropsical swellings, and can say that I shall always prefer it to anything else, when I can procure it. And I believe that it is valuable in any form of dropsy, without reference to its cause or locality.

In one case a woman was attacked by measles immediately after a confinement, and in recovering she had anasarcous swelling of the feet and legs up to the knees, so that she almost lost the use of her lower limbs. The recovery was very prompt and complete, under the use of a decoction of the chimaphila. Another case, a man similarly diseased with anasarca of the lower extremities, was also promptly and effectually relieved. I used it as follows: An ordinary handfull of the herb (root, stem and leaves altogether) to about one gallon of water, and boiled to half a gallon. This should color the water very considerably—give a black tinge to it. Of this decoction one pint should be taken every day.

I do not think that pipsissewa has any dangerous qualities whatever. I refer the reader to Dr. Porcher's book, "Resources of the Southern Fields and Forests," for further information about this plant. It is undoubtedly one of our most valuable and reliable resources, and deserves more attention than it has received. I know a physician who says that he has used a thousand gallons of the decoction, which would indicate good service in his hands.

I do not know whether the *sisyrinchium bermudianum* has any common name or not, but it grows in every part of Georgia, and very abundantly in some places. It has no deleterious properties, and, for the purpose for which I recommend it, I believe it has no superior in the materia medica. It is the most reliable cathartic I have ever used. And while entirely reliable, it is one of the mildest and gentlest purgatives that I know. I have used it successfully in cases which have resisted calomel, oil, and epsom salt, given one after the other. The operation which it produces is not colliquative, nor attended with straining, but is much like a natural operation when the bowels are somewhat loose. The mildness of its effects and its thorough reliability, give it a high place in my estimation. A moderate handfull of leaves put into a cup, and the cup then filled with boiling water, prepares the tea for use on cooling. The cupful should be taken as the dose. It is an additional recommendation that the infusion thus prepared, may be sweetened, if desired, with sugar, to the taste, especially for children.

The next plant I shall notice is *saururus cernuus*, known to some as the swamp lily. It also bears the name of lizzard tail. I do not think it is found common in Georgia much above the pine belt which crosses the State from Columbus to Augusta, although I have seen it in Polk county. Its natural habitat is the low, marshy lands of the lower portion of the State.

The saururus has a root possessing the characters somewhat of a sub-ærial creeper, being nodular, and giving off rootlets at each node or joint. Pulpified and made into a poultice, or prepared as an ointment, it is a superior application to any kind of ulcer, sore or sprain. During the late war, an officer of the regiment to which I was attached, was thrown from a buggy, and falling with his whole weight upon one of his outstretched hands, sprained his wrist-joint so badly that he could not use it at all, on account of the pain produced by the least motion. He applied to me for something to relieve it. Having nothing on hand at all suitable, I resorted to this root. I had some gathered and a poultice made. It was applied in the afternoon (the accident occurred in the forenoon) and the next morning he could use the injured hand equally with the other and it gave no further trouble.

Some years since I was in the country a few miles south of Atlanta, and, in conversation about plants and their uses, I related the above circumstance, giving the botanic name of the plant, not then knowing any other. An old farmer present then stated that a little daughter of his had at one time injured her hip in some way, so that it produced a running sore, which discharged a teacupful of pus every day, and continued doing so for a long time. Everything else failing, he was induced to try a poultice made of the root of a plant which was abundant where he then lived, (about forty miles below his present home) and he stated that the sore was completely healed up in about one week, and has remained so. He added that he was so well pleased with its effects, that when he moved to his home he brought some of the seed with him, and planted them near his present dwelling. This case being so pointed and plain, I wished to see the plant, and went to where it was growing, when I recognized it as my own plant saururus. We had both been all the time talking of the same plant under different names. He called it swamp lilly, which name is a very pretty one, but not appropriate, as the plant is not a lily at all, and has very few characters in common with the lily class of plants. But there is no ground to quarrel with popular nomenclature, as, after all, it is probably almost as rational as that of the regular scientific botanist.

The genus of plants called *salvia*, derives its name from the Latin *salveo, to save, to heal,* and is represented in our gardens by the common sage. The name thus derived indicates that since the days of Linnæus, who bestowed the name upon it, it has been recognized as possessing healing virtues. There are *ten* species of salvia in the United States. The *salvia lyrata* is quite abundant in Middle Georgia, and extends to the coast. I have found it near Atlanta, but it is rare here, I think, and will scarcely be found above here.

The *salvia lyrata* is valuable in similar cases with the swamp lily, and in domestic practice has proven eminently useful and valuable as a poultice in carbuncle. The entire plant is used. It should be pulpified and applied so as to cover the entire carbuncle. This plant and the loblolly bay (*gordonia lasianthus*) would make an excellent combination in an ointment—to be made from the extracts. Or an extract from each, used either separately or in combination, rubbed up with lard or tallow, would make, I believe, one of our best applications to any form of solution-of-continuity of the soft tissues.

The loblolly bay is found in the coast counties of Georgia and Florida, but extends some little distance inland, along the banks of the rivers, especially when their margins are low and swampy.

